# FPGA-Based Soft-Core Processors for Image Processing Applications

**DOI:** 10.1007/s11265-016-1185-7

**Published:** 2016-10-10

**Authors:** Moslem Amiri, Fahad Manzoor Siddiqui, Colm Kelly, Roger Woods, Karen Rafferty, Burak Bardak

**Affiliations:** 1 School of Electronics, Electrical Engineering and Computer Science, Queen’s University Belfast, Stranmillis Road, Belfast, Northern Ireland UK; 2 Thales, Belfast, Northern Ireland UK; 3 Department of Computer Engineering, European University of Lefke, Gemikonagi, Turkey

**Keywords:** Image processing, FPGAs, Heterogeneous multi-core architecture

## Abstract

With security and surveillance, there is an increasing need to process image data efficiently and effectively either at source or in a large data network. Whilst a Field-Programmable Gate Array has been seen as a key technology for enabling this, the design process has been viewed as problematic in terms of the time and effort needed for implementation and verification. The work here proposes a different approach of using optimized FPGA-based soft-core processors which allows the user to exploit the task and data level parallelism to achieve the quality of dedicated FPGA implementations whilst reducing design time. The paper also reports some preliminary progress on the design flow to program the structure. An implementation for a Histogram of Gradients algorithm is also reported which shows that a performance of 328 fps can be achieved with this design approach, whilst avoiding the long design time, verification and debugging steps associated with conventional FPGA implementations.

## Introduction

The emerging need for processing big data-sets of high-resolution image processing applications demands faster, configurable, high throughput systems with better energy efficiency [8, 17]. Field-Programmable Gate Arrays (FPGAs) can play an important role as they can provide configurability, scalability and concurrency to match the required throughput rates of the application under consideration [27]. They have the potential for distributing image processing to a computing platform which is located as close as possible to the image source. This distributed processing can act to reduce the need for bandwidth and power on a large scale, which in turn reduces the communication overhead and the amount of data needed to be stored.

Typically FPGAs work well with the applications which require concurrency, high bandwidth and re-programmability. However, FPGA design and verification is time-consuming and requires that designers create system implementations using Hardware Description Languages (HDLs) such as VHDL and Verilog [6]. The HDL approach allows a digital circuit to be precisely described, and with timing constraints met, the design tools can then synthesise, map, place and route the HDL design accordingly. The major issue is that this design process involves numerous verification and debugging steps, which increases the time to market from weeks to months, depending on the complexity of the algorithm of interest [10]. In order to reduce the required design time and effort, the two biggest FPGA vendors, Xilinx and Altera, have created new High Level Synthesis (HLS) tools which allow the designer to use high level languages such as C [11] or OpenCL [25] to create algorithmic representations for FPGA implementation. There are also other high level synthesis routes reported in open literature.

All of the HLS design tools, however, still rely on the HDL synthesis route to produce the programming files, so a synthesis and implementation route still has to be performed for the targeted technology which can take up to several hours. Moreover, every time a design change is performed, this process has to be repeated. This paper proposes an alternative approach based on developing a highly efficient, RISC (Reduced Instruction Set Computing) processor called Image Processing PROcessor (IPPro) [24]. The bespoke designed soft processors have guaranteed performance and resource usage; they are also easily reprogrammable and even allow potential support for run-time reconfigurability. The proposed approach uses the CAL dataflow language approach [14], providing a design route to allow the user to decompose their design into a series of small actors which allow the user to exploit task and data parallelism existing in the algorithm [2, 3] and which can then be compiled to IPPro architectures.

The novel contributions of the paper are:

- Overview of the IPPro processor which has been optimised to match both the image processing algorithms requirements and FPGA resources and which avoids the need for long place and route implementation;
- Creation of a multi-core architecture with an inter processor communication network which is targeted for complex image processing systems;
- Development of a dataflow system based on the CAL language which provides a route for users to produce code for the processors;
- Profiling and implementation of a complex image processing application namely, the Histogram of Oriented Gradients (HOG) algorithm.

The paper is organized as follows. Section 2 reviews related background work in the area of existing soft-core processors and some information on dataflow languages and tools, in particular, the RVC-CAL language. Section 3 briefly outlines our proposed, many-core, heterogeneous architecture for implementing image processing applications. Section 4 describes the proposed dataflow framework and how the programming paradigm is achieved. Section 5 presents a detailed case study for a HOG design example implemented using the design framework and using soft-processor architecture, for which a performance comparison is also made. Section 6 concludes and reviews the proposed approach.

## Background

The reprogrammable design methodology proposed in this paper removes the requirement for HDL entry, synthesis, and place and route processes. The approach replaces the reconfigurability property of FPGAs by a reprogrammable model. In order to do this, an intermediate fine-grained reprogrammable architecture is proposed which involves programmable, multi-core processors and an associated communication network. The proposed system consists of RISC architectures which support Single Instruction Multiple Data (SIMD) operations, and various inter-processor communication methodologies, to provide the required flexibility and programmability. This reprogrammable archiecture has been designed to be as compact as possible to increase the efficiency of the use of the available FPGA logic whilst also achieving high performance [24].

In this concept, if every single processor can be thought of as an actor and between the actors data is fired through the First In, First Out (FIFO) structures, the overall system would suit the application domain and would be highly applicable to model and program through a dataflow language and framework. Dataflow languages in general have the ability to express the parallelism, and also make it easy to identify and resolve data dependencies to exploit concurrency as much as possible. However, since an FPGA-based platform is targeted with given limitations such as restricted memory, a dataflow language-based framework should consider these issues.

### Soft-Core Processors

There are a number of state-of-the-art, soft-core processors based on FPGA architectures. These include FlexGrip [1], IDEA [7], and DSP48E-based MIMO (Multiple Input Multiple Output) processor [9]. FlexGrip maps pre-compiled CUDA kernels on soft-core processors which are programmable, flexible and scalable and which can operate at 100 MHz. The IDEA processor and MIMO processor have a similar structure to our IPPro core discussed here, as both use the Xilinx DSP48E1 processing unit as their Arithmetic Logic Unit (ALU). The IDEA processor uses an 8-stage pipeline to achieve 407 MHz clocking frequency, and MIMO processor supports a very specific instruction set for Multiple Input Multiple Output (MIMO) communication systems and is able to work at a clock frequency of 265 MHz.

Given the starting point of using a high-level, dataflow language, there are a number of challenges behind creating an efficient implementation. These include the control of memory size, throughput and bottleneck analysis. The main advantage for using an FPGA as a target implementation platform is the high bandwidth, low latency memory access which increases the throughput of the applications of interest. On the other hand, whilst the availability of multiple memories is attractive from an image processing implementation perspective, the overall memory is limited particularly when compared to competing technologies such as GPUs.

**Figure 1:**
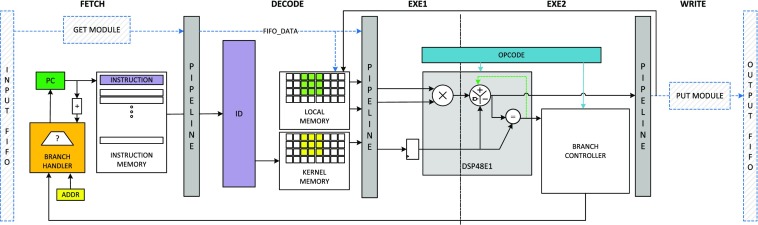
IPPro architecture.

The IPPro is our hand-coded RISC soft-core processor. By using the Xilinx DSP48E1 primitive as an ALU and minimizing supporting logic, synthesis results show that it is capable of running at 526 MHz on Xilinx SoCs using an XC7Z020-3 [24]. It uses one DSP48E1, one BRAM and 330 Slice Registers (excluding input/output FIFOs) per processor. IPPro outperforms all other current FPGA based soft-core solutions as it has been optimised for modern FPGA technologies and provides a good balance between processing elements and memory. It supports various instructions and memory accesses and is capable of processing signed 16-bit operations. The IPPro processor architecture uses a 5-stage balanced pipelining and supports streaming mode operation where the input and output data is read and written back to FIFO structures, as shown in Fig. 1. This processor is designed to be compact, reprogrammable, and scalable to achieve high throughput rates which are comparable to custom-made HDL designs.

IPPro keeps the balance between programmability and the need to maintain the FPGA performance. Overall, it has the following addressing modes:

- Local Memory – Local Memory (LM-LM)
- Local Memory – FIFO (LM-FIFO)
- Kernel Memory – FIFO (KM-FIFO)

The local memory is composed of general-purpose registers used mainly for storing operands of instructions or pixels. This memory currently contains 32 16-bit registers. A FIFO is a single internal register of IPPro where the input and output streams from/to an external FIFO are stored. Kernel memory is a specialized location for coefficient storage in windowing and filtering operations with 32 of 16-bit registers.

An example of the supported instructions can be seen in Table 1. This table shows the IPPro LM-FIFO addressing mode instructions and some miscellaneous ones among others. The IPPro instruction set is capable of processing basic arithmetic and logical operations for different addressing modes. In addition to the unary and binary instructions, it also has support for trinary expressions such as MULADD, MULSUB, MULACC and others. Given the limited instruction support and requirements from the application domain, a coprocessor is added to provide better support for more complex processes such as division and square root. A more detailed description of the IPPro is given in references [18, 24].

**Table 1:** Example IPPro instructions.

LM-FIFO		Misc	
ADD	LOR	JMP	GET
SUB	LNOR	BNEQ	PUSH
MUL	LNOT	BEQ	NOP
MULADD	LNAND	BZ	BYPASS
MULSUB	LAND	BNZ	
MULACC	LSL	BS	
LXOR	LSR	BNS	
LXNR	MIN	BNGT	
	MAX	BGT	

### Dataflow Languages and Tools

The dataflow representation concept was introduced by Sutherland [26] as a way to describe arithmetic operations. The graphical representation of the arithmetic operations makes it easier to distinguish the temporary variables, dependencies and input and output variables, and most importantly here, data transfer rate between the processing elements, i.e. actors. Dennis et al. [13] formally described the concept of directed graphs with the flow of data between edges of actors. A dataflow program consists of actors and its firing rules, where every actor describes the required arithmetic/mathematical operation to process the input streams before passing the result(s) to the output streams. The representation of actors in dataflow programming models are given by directed graphs where the nodes represent computations and in general, the arcs represent the movement of data.

The main principles behind the dataflow design methodology are the concurrency, scalability, modularity and data-driven properties. The term data-driven is used to express the execution control of dataflow with the availability of the data itself. In this context, an actor is a standalone entity which defines an execution procedure. Actors communicate with other actors by passing data tokens, and the execution is done through the token passing. The combination of a set of actors with a set of connections between actors constructs a *network*. Within the defined networks, communication is made using infinite size FIFO components.

In summary, a dataflow program is defined as a directed graph of computational elements communicating through ports. Since Sutherland’s proposition, dataflow programming has been studied in detail and various languages have been proposed for different target applications. *Lustre* [16] is a synchronous dataflow language developed for programming real-time systems and is used within an industrial embedded software toolset called SCADE. *Signal* [19] is a synchronous dataflow language and its compiler is developed for safe real-time system applications. Its semantics are defined for multiple-clocked flows of data and events. The *MAPS* framework concentrates on mapping multiple dataflow applications onto heterogeneous MPSoCs using design constraints for performance estimation and mapping. *Ptolemy II* is an open source dataflow system design environment based on an actor-oriented design. It supports process networks (PN), discrete-events (DE), synchronous dataflow (SDF), synchronous/reactive (SR), rendezvous-based models, 3-D visualization, and continuous-time models. CAL [14] has been developed for image processing and used for FPGAs, hence it seemed a highly appropriate choice for the approach proposed here and is described in detail next.

### RVC-CAL Dataflow Language

CAL [14] was developed by Eker and Janneck as a part of the Ptolemy II project and is a high-level programming language for writing actors where within these actors, input streams are transformed to output streams. CAL offers the necessary constructs for expressing parallel or sequential coding, bitwise types, a consistent memory model, and communication between parallel tasks through queues. The CAL computation model enables the programmer to express applications as network processes making it an ideal candidate to be used as a behavioural description for modeling software and hardware processing elements. A subset of CAL language is called Reconfigurable Video Coding CAL or RVC-CAL where type limitations are applied and advance features of CAL language are prohibited; it is the language used in Orcc which is an open source dataflow development environment and compiler framework, that allows the transcompilation of actors and generates equivalent codes depending on the chosen back-ends [28].

The RVC framework is a standard originally developed for MPEG in order to provide a unified, high-level specification of current and future MPEG video coding technologies using dataflow models. In this framework, a decoder is generated by configuring video coding modules which are standard MPEG toolbox or propriety libraries. RVC-CAL is used to write the reference software of library elements. A decoder configuration is defined in the XML language by connecting a set of RVC-CAL modules.

In general, an RVC-CAL based design is composed of several files. The file types and their contents are as follows.

- Dataflow network (.xdf file): this is a textual description coded in .xml format constructing the network of actors of the design and the flow of data between them.
- Actors (.cal files): an actor processes a stream of tokens received through its input ports and sends the processed tokens through its output ports. A design can have multiple actors connected to each other as specified in the dataflow network file. The basic structure of an actor includes the input/output ports and one or more actions. An action will be executed, i.e. fired, if all the following activation conditions are met:
  - All required input tokens are available;
  - The guard expression holds true;
  - No other action with higher priority can be activated at this time;
  - The action can be fired based on the action schedule.
- Data file (.cal file): this is a special kind of .cal file to define constants. These constants can be imported into and used by any actor in the design.
  A typical structure of an actor code containing two actions is shown in the following.

**Figure:**
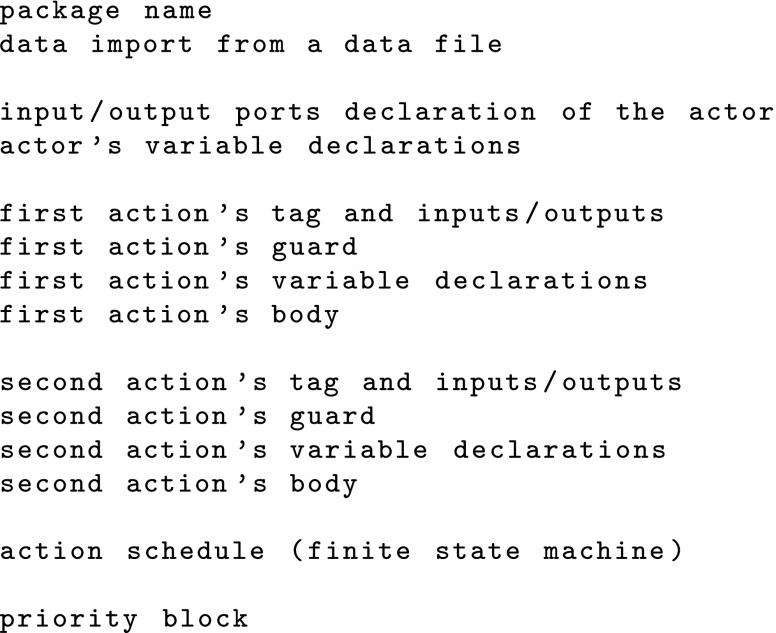

## Many-Core Heterogeneous Architecture

This section describes the proposed heterogeneous architecture for the implementation of data-intensive, streaming-based applications. This design has been focused initially towards the All Programmable System on Chip (AP SoC) devices, in particular, the Xilinx Zynq-7000 AP SoC. These devices integrate a Processing System (PS) and a Programmable Logic (PL) portion in a single device. This work makes use of both the PS and PL portions; some controlling applications and potentially some actors (depending on the decision on software/hardware partitioning, as will be described later) execute on PS, and the compute-intensive actors are realised on a network of multi soft-cores implemented on PL.

### Inter Processor Communication Network

In programmable multi-core architectures, the data communication architecture chosen to exchange data among different cores is important, and the design choices made can significantly impact system performance. With the use of an inter processor communication network, the range and complexity of the targeted applications will increase. In most cases, adaptive algorithms running on multi-core systems need to communicate with other cores to fulfill the required memory and execution semantics.

From a hardware perspective, it provides flexibility, scalability and bandwidth whereas from the software perspective, it defines what applications could efficiently map and schedule on the underlying architecture [4]. In the case of programmable architectures, the mapping and scheduling of the application is realised during decomposition and compilation which means that the underlying architecture has direct implications on the framework development.

Image processing applications exhibit structures for different execution and memory access patterns [4, 22, 23], some of which are classified in Table 2. These are mainly algorithmic characteristics, and hence are platform independent and equally valid for different computing platforms CPU, GPU, FPGA etc.. These patterns can give an idea about the level of connectivity, memory, scheduling and mapping requirements of an application. If a certain type is supported by the underlying architecture, it would be able to run most of the algorithms that are similar within the respective class.

**Table 2:** Execution and memory access patterns [22].

Type	Memory Pattern	Execution Pattern
Pixel-Pixel (P2P)	Pipelined	One-one
Neighbour-Pixel (N2P)	Coalesced	Tree
Reduction to Scalar (R2S)	Recursive Coalesced	Reduction Tree
Reduction to Vector (R2V)	Recursive Non-Coalesced	Large Tree Reduction

The ideal architecture is one that could support mapping and scheduling of the mentioned patterns in order to allow the ultimate goal of the IPPro architecture which looks to accelerate a wide range of image processing applications. These patterns have been implemented using the stream- and dataflow-based computing paradigm. The design choices have been driven from both sides of the project flow, i.e. top to bottom (starting from high-level description) and bottom to top (starting from physical resources placement). This drives the development as follows:

- Multiple to multiple core level connectivity;
- Area utilisation in terms of underlying FPGA logic;
- Maintenance of the balance between memory and bandwidth;
- Maintenance of the critical path length to ensure high performance.

**Figure 2:**
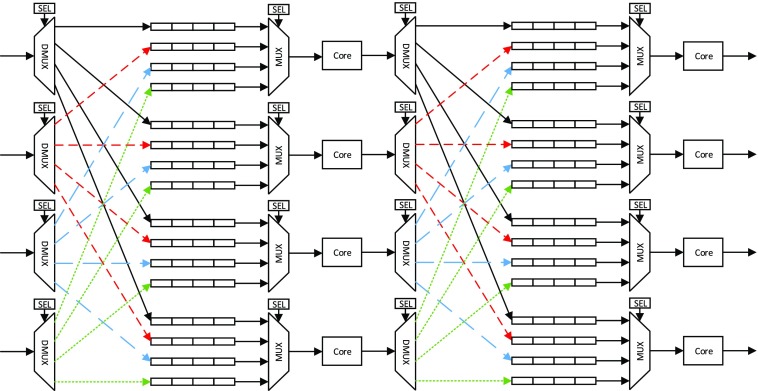
Interconnection network of an array of cores.

The proposed multi-core processing network is currently implemented as an array of 4×*C* soft cores, where the number of columns (*C*) is decided based on the available resources of the target device. This architecture has evolved from a detailed analysis of mapping a series of algorithms onto a multi-core architecture. Figure 2 shows a simplified basic unit of a network constructed as the interconnection of 4×2 cores. The larger network can be formed by extending and replicating this unit from either side. Every core can control one multiplexer (mux) and one demultiplexer (demux). The mux is connected to the input port of the core where one of the four FIFOs can be selected to pass its tokens to the core. Each FIFO is connected to every core of the previous layer hence providing full connectivity. The core is also in control of a demux connected to its output port. Through this selection, it could send the partially-processed tokens to any of the four cores located in the following layer of the network.

The basic streaming-based operations for a core in such a network is as follows:

1. An action in RVC-CAL will be fired only if all the required tokens are available. The core will wait until all these tokens are received from the expected source cores through the input port.
2. With all the tokens available, the core will process the tokens.
3. Every processed token will be sent through its output port and shifted in the connected FIFO of the destination core which expects this token.
4. This process will be repeated as long as the streaming-based application keeps running.

For the above network to run, selection lines of muxes and demuxes should be set correctly by the cores and access to the FIFOs should be coordinated. Also the order of the tokens passed through FIFOs should be preserved. Such settings allow the architecture to be optimised from an application specific perspective and then not changed. This requires extra information about the connectivity of the streaming based network, and orderly passing of the tokens. These issues are dealt with during the compilation process of a specific application, as will be explained later.

### System-Level Design

**Figure 3:**
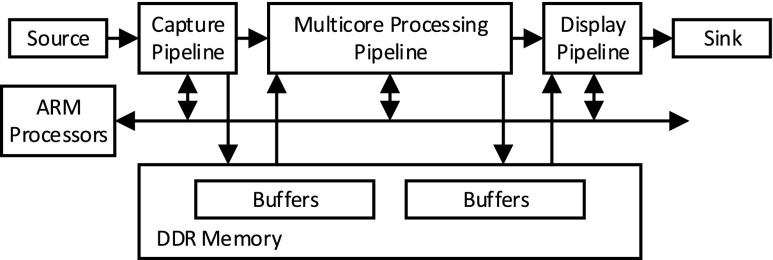
Streaming pipeline architecture.

Our initial implementation target technology for streaming-based video processing applications is the Zynq-7000 AP SoC. This device integrates the software programmability of a dual-core ARM Cortex-A9 based PS and the hardware programmability of PL. In general, our design involves mapping the data flow control onto the PS (ARM cores), and image-processing application on the multi-core processing data path realised on PL to achieve real-time processing of compute-intensive applications. It might be the case that the full image-processing application is not realised on PL as less computational demanding functions often only require memory re-organisation. Implementing such functionality at the PS level could be the most efficient method as it could avoid passing large volumes of data between PL and the ARM cores, thereby avoiding costly transport delays. In general, the streaming pipeline architecture is designed as represented in Fig. 3; the PL implements a video design that consists of a capture pipeline, a multi-core processing network, and a display pipeline.

**Figure 4:**
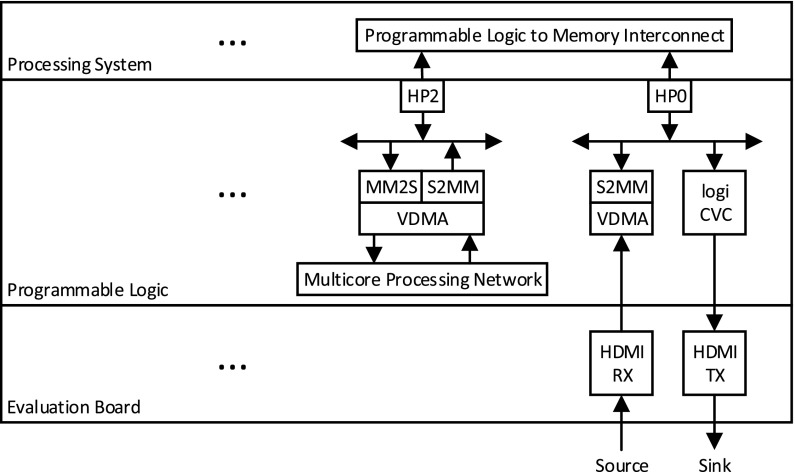
A portion of system-level block diagram of PL and PS of the design.

A part of the system-level block diagram of this design, which uses Xilinx proprietary IPs available for Zynq SoC devices, is illustrated in Fig. 4. The capture pipeline includes a VDMA with one write channel and is connected to the HP0 write port. The VDMA writes the incoming video frames through an HDMI receiver into buffers inside the memory. The multi-core processing network is connected to the HP2 read/write ports. A VDMA (with one read and one write channel) reads pixels from memory and sends them to the multi-core network for processing. The VDMA writes the processed data back into memory through its write channel. The display pipeline is connected to HP0 read port. This pipeline consists of the logiCVC display controller which has an integrated DMA engine to read buffers from memory and send the data to the monitor over HDMI.

## Dataflow Framework

This section describes the proposed tool flow, concepts and techniques for the implementation of image processing applications, described in RVC-CAL dataflow language, on AP SoC devices.

**Figure 5:**
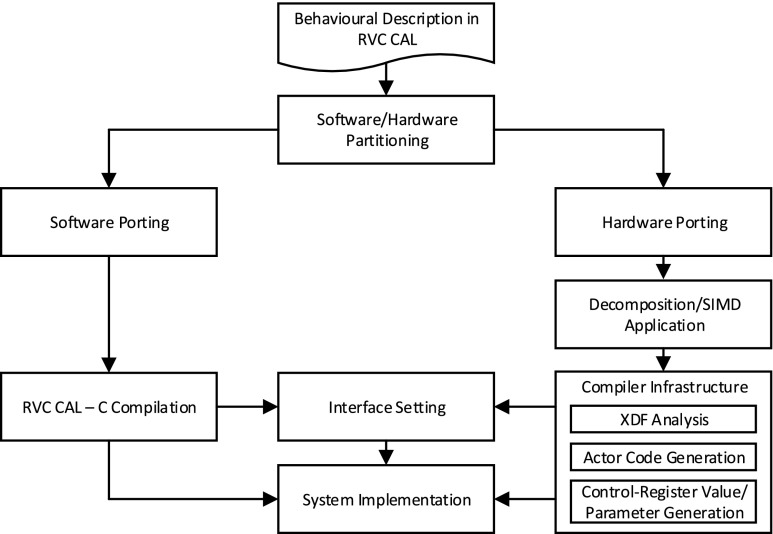
Simplified design flow of a hardware and software heterogeneous system.

Our developed tool flow for the implementation of image processing applications is shown in Fig. 5. The input to the framework by the user is the behavioural description of an image-processing algorithm coded in the RVC-CAL dataflow language. This behavioural implementation is expected to be composed of multiple actors along with an xdf dataflow network description. Some of these actors are selected to execute in soft-cores (one actor per core) hence providing concurrent execution of these actors, and the rest to run in the host CPUs. By analysing the behavioural description of the algorithm, the software/hardware partitioning of the design is determined. The metrics involved in this decision-making will be discussed later.

Once the actors are split based on their target execution platform, the original xdf file no longer represents the network topology of either of the two sets. Each set of actors should be redesigned separately and their input/output ports fixed and each set’s xdf dataflow network description file generated. This can easily be done using the Orcc Development Environment.

The actors to run on the host CPUs are compiled from RVC-CAL to C using the C backend of Orcc Development Environment. The actors to be accelerated using the proposed IPPro-based multi-core network are first analysed for decomposition and/or SIMD application, and then passed through a compiler framework. Both of these important steps will be discussed later. The compilation flow is composed of three distinctive steps. The first step investigates the xdf dataflow network file and assigns the actors to the processors on the network and keeps a record of the settings for each actor to communicate with the other ones to establish the data streams. The second step of the compilation is the conversion of each actor’s RVC-CAL code to IPPro assembly code. The final step is the generation of control register values, mainly for AXI Lite Registers, and parameters required by the developed C-APIs running on the host CPUs.

While the interconnects and input/output ports ‘between’ the FPGA-targeted actors are handled by the compiler, receiving image data by the first-level actors and sending the results from the final-level actors back requires some development work and creation of settings. Multiple controllers (programmable by the host CPUs) are designed to provide the interface to transfer the image data to the accelerators and gather the results and transfer back to the host. This part of the design is currently custom-designed or manually handled in our implementation. The fully-programmable implementation is a subject for future work.

With the host CPUs running part of the design and setting control registers and C control functions parameters, the IPPro binary codes of the other actors loaded to the proper cores on the accelerator, and the interface between the software/hardware sections set accordingly, the system implementation is in place and ready to run.

### Software/Hardware Partitioning and Decomposition of the SIMD Application

An initial version of a performance analysis tool or profiler has been developed and embedded in the partitioning and decomposition tools in order to evaluate how well the decomposed actors will perform on the new architecture. Various static and dynamic profiling tools and techniques exist in open literature, such as that in Simone et al. [5] who proposed a very beneficial design space framework for profiling and optimising algorithms which also works with the Orcc development environment. This profiler is built to work with HLS-based designs and is not applicable to our processor-based approach. To develop a profiler for our framework, a cost model i.e. a set of metrics has been created as a means of quantifying the effectiveness of the decomposition and mapping of actors to the IPPro network architecture. To realise the cost model, architectural parameters/constraints which should be satisfied to achieve high-performance and area-efficient implementations need to be identified and a method needs to be determined which can quantify the identified metrics for performance/area measurements by a profiler.

For a many-core heterogeneous architecture, the metrics/constraints which are the deciding factors in the partitioning/decomposition process can be categorised as ‘performance-based’ and ‘area-based’. The important performance-based metrics are implemented and discussed here. The area-based metrics are a subject for future work and will be briefly discussed later. The three performance factors to be considered are:

- *Actor execution time* which is the main factor affecting performance and can be estimated from the actor’s code. To find the exact execution time of an actor, it needs to be compiled first and its instructions counted. The actors with the longest delays which are parallelisable are suitable for acceleration.
- *Overheads incurred in transferring the image data to/from the accelerator* also affect acceleration performance. If an actor requires the entire image to be available for processing or it produces large amount of data to be transferred to the host CPUs, the performance will probably improve by executing it in the host CPUs.
- *Average waiting time* is that needed to receive the input tokens and send the produced tokens to another actor, although this could be included in the actor’s execution time.

**Figure 6:**
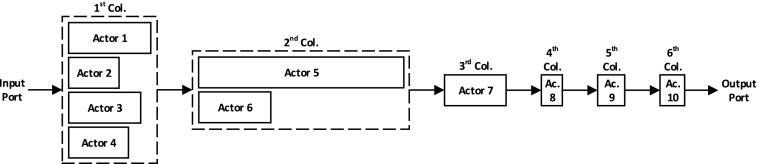
Dataflow block diagram of an example design consisting of 10 actors organised in 6 columns. The width of every actor is representative of its execution time.

**Figure 7:**
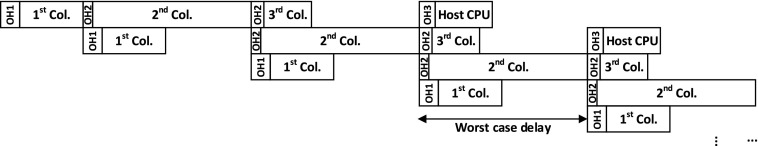
A section of the pipeline showing the impact of actor execution times on the performance of the design of Fig. 6. The three types of overhead for data transfer/distribution to accelerator, data transfer between cores in the accelerator, and data collection/transfer from accelerator are represented respectively by OH1, OH2, and OH3.

Given a dataflow network of a design such as the one shown in Fig. 6 where actors’ execution times are reflected in their shapes, the performance can be analysed by considering its pipeline execution structure. This design has a total of 10 actors arranged in 6 columns where the number of cores in every column varies between 1 and 4. A section of the pipeline of this design is shown in Fig. 7. The three communication overheads considered are:

- Overhead to transfer data from host CPUs to the accelerator and then distribute among cores (OH1);
- Overhead to transfer data between actors through FIFOs (OH2) and;
- Overhead to collect the processed data and transfer it back to the host CPUs (OH3).

Using this diagram, a main image processing performance metric, frames/s (fps), can be approximated considering the following features (along with the abbreviation of each feature):

- D: the worst case delay (execution clock cycles) of all the stages (columns);
- P: number of pixels in a frame;
- C: number of pixels consumed on every pass;
- F: hardware clock rate.
  1 $$ fps \approx \frac{F}{D \times \frac{P}{C}} $$

In this calculation, the average overhead of the longest actor is included in its execution time. This overhead can usually be ignored since the actor with the longest delay should have its input tokens ready by the shorter actors which are quicker. Considering Eq. 1, it can be concluded that to improve the fps, we need to:

- Increase SIMD operations, by generating multiple instances of the original design, using the same instruction memory for the corresponding instances and providing appropriate data distribution and collection controllers. This will decrease $\frac {P}{C}$.
- Decrease the execution times of cores by decomposing them; this will increase the number of columns in the design and hence the degree of parallelism will increase. In the equation, this will result in decrease of *D*. In Figs. 6 and 7, the decomposition of the actor with the worst-case delay in 2 ^nd^ column will improve the performance.

The host CPU could be considered as one stage of the dataflow and since its clock rate is higher than that of FPGA, the assigned actor’s execution clock cycles could be higher to run in parallel with the shorter actors executing on FPGA. If multiple short actors (compared to the average execution time expected to satisfy the required performance) are sequentially placed in the dataflow, they can be merged to reduce the overhead of token transfer through FIFOs and also reduce area utilisation as less cores will be used up by the design. If these short actors are placed at the start or end of the flow, they are the best candidates to be partitioned for execution in the host CPU. The three final short actors in Fig. 6 are merged and running in the host CPU, as indicated in Fig. 7. If placed in the middle of the dataflow, the cost of transmission to host CPU and then back to the FPGA will typically be high and it would be better to accelerate it.

The behavioural description of an algorithm could be coded in different formats:

- No explicit balanced actors or actions are provided by the user.
- The actors include actions which are balanced without depending on each other, e.g. no global variables in an actor is updated by one action and then used by the other ones. These actions need to be decomposed into separate actors.
- The actors are explicitly balanced and only need to be partitioned into software/hardware execution.

There are two different types of decomposition: ‘row-wise’ and ‘column-wise’. In row-wise decomposition, there is no dependence among newly-generated actors while in column-wise decomposition, the new actors are dependent on each other. The first case mentioned above will most likely result in column-wise decomposition and the second one in row-wise. The row-wise implementation is preferred over column-wise, as in row-wise no overhead of token transmission is incurred compared to the column-wise where this overhead could be a limiting factor in the decomposition process. A combination of these two can also be implemented in certain conditions.

If the actors or actions are not balanced, a number of steps should be taken to decompose it. The main step is to find the basic blocks of the code. A basic block is a sequence of instructions without branches, except possibly at the end, and without branch targets or branch labels, except possibly at the beginning. The first phase of decomposition is breaking the program into basic blocks. Some examples of basic blocks in RVC-CAL are: If statement, while statement, foreach statement, and assignments. Then the ‘balance points’ of the actor should be found. The balance points divide the actor into multiple sets of basic blocks such that if each set is placed in a new actor, the overhead of transferring tokens among the sets will not create a bottleneck and the performance requirements of the algorithm will be satisfied. There could be more than one balance point available for grouping basic blocks in which the one with lower overhead should be used.

**Figure 8:**
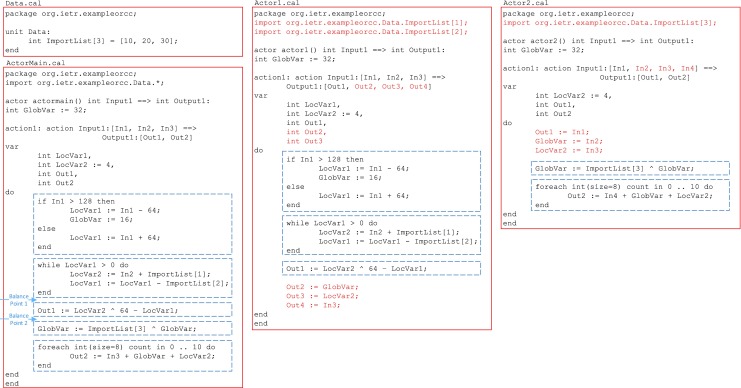
An example column-wise decomposition process where ActorMain.cal is decomposed into Actor1.cal and Actor2.cal. This example is only for demonstration purposes. The basic blocks are shown in dashed boxes and the changes are highlighted in colour.

Figure 8 shows an example actor ActorMain.cal which does not meet the required performance and should be decomposed. The basic blocks of the actor are highlighted in this code. There are two balance points which satisfy the performance requirements; since either of them divides the code into two sets of basic blocks where the second one is dependent on the first one, this is a column-wise decomposition. A balance point should be chosen which reduces the token transmission through FIFOs; balance point 1 requires one extra token (LocVar1) compared to balance point 2. Therefore balance point 2 is the better choice.

**Figure 9:**
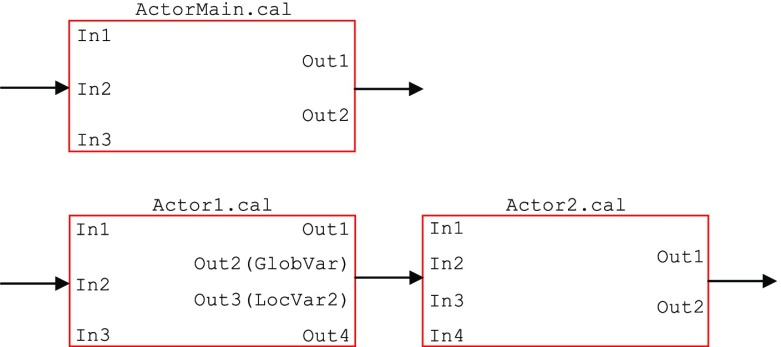
Decomposition impact on the input/output ports of the example shown in Fig. 8.

A disadvantage of column-wise decomposition is that the required unprocessed tokens by an actor should pass through the preceding actors (for example Out4 := In3; assignment in Actor1.cal of Fig. 8), and the processed output tokens produced by first-layer actors should be passed through the following actors (for example Out1 := In1; assignment in Actor2.cal of Fig. 8). This adds to the token transmission overhead of the design. Column-wise decomposition, however does not need any changes to be made to the ports of surrounding actors. The communication overhead for the example of Fig. 8 is shown in Fig. 9.

**Figure 10:**
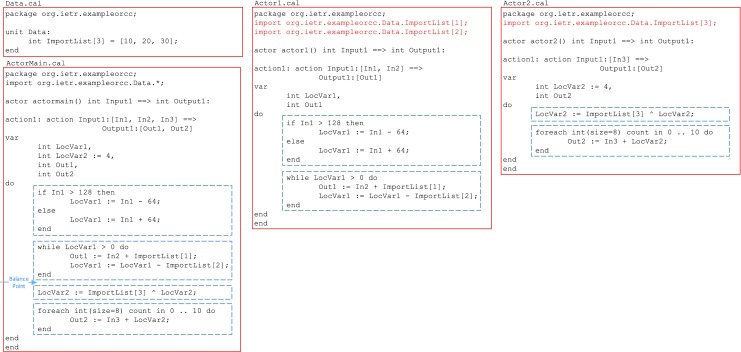
An example row-wise decomposition process where ActorMain.cal is decomposed into Actor1.cal and Actor2.cal. This example is only for demonstration purposes. The basic blocks are shown in dashed boxes and the changes are highlighted in colour.

**Figure 11:**
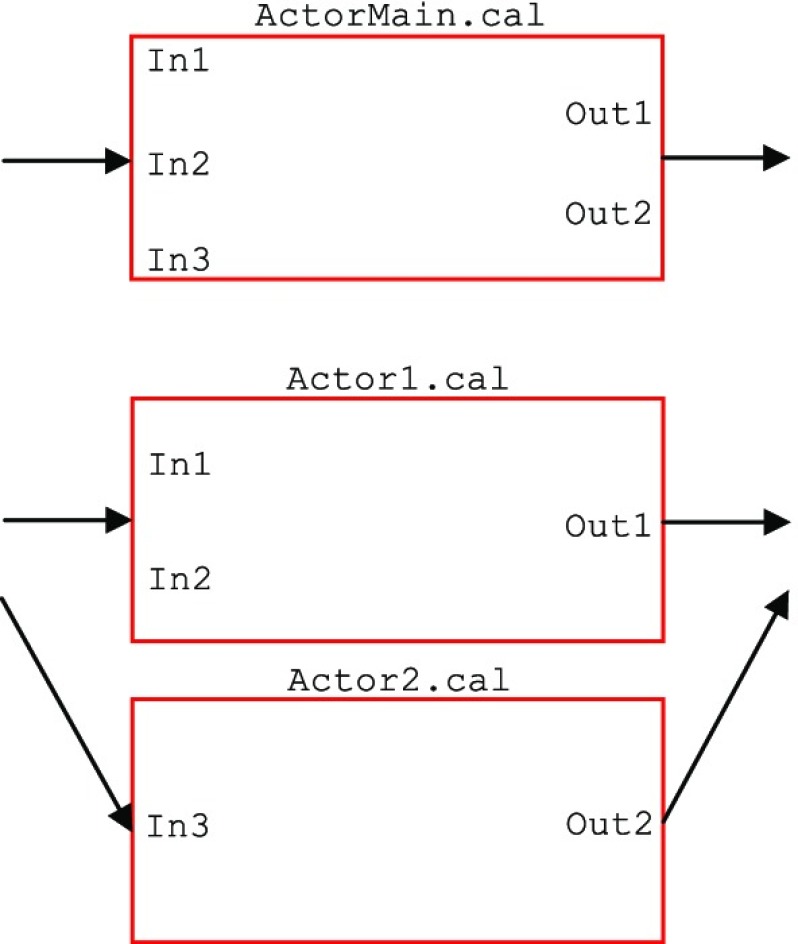
Decomposition impact on the input/output ports of the example shown in Fig. 10.

If an actor includes actions which are balanced and independent of each other (with a linear scheduling), or equivalently, the basic block sets inside ‘one’ action are independent of each other around the balance point, the row-wise decomposition can be applied. In the example shown in Fig. 10, ActorMain.cal has two independent sets of basic blocks around the balance point, hence row-wise decomposition can be applied. This type of decomposition does not increase the token transfer overhead when compared against the original actor; it only changes the ports through which the tokens are communicated with the adjacent actors in the dataflow graph; the connecting ports of the neighbouring actors should change to fit the new structure. Figure 11 shows the decomposition impact on the port declarations of this example.

#### Metrics

As mentioned earlier, the classification of metrics involved in partitioning/decomposition is performance-based or area-based. In our implementation, we have considered the main system-level performance-based features, however, there are more metrics involved and the important ones are reviewed in this section. Some of these metrics are currently manually checked in our design, and automatic application of them will be in our future work. For a many-core heterogeneous architecture, the metrics/constraints involved in the partitioning/decomposition process can be categorised as core-level, network-level, and system-level ones. The important metrics of each level are discussed in the following and summarised in Table 3. To simplify the design process and multi-core network, every decomposed actor is limited to containing one action and being mapped to one soft-core.

**Table 3:** Important metrics used in decomposition phase

Level	Metric	Max	Changes will mainly affect:
Core	Actor’s no. of instructions	1000	Peak register usage
	Actor’s average execution time	—	Token prod./cons. rate
	Core code efficiency	<100 %	Average degree of concurrency
	Peak register usage	32	Actor’s no. of instructions
	Core bandwidth	—	Core code efficiency
Network	Core utilisation	100 %	—
	Token prod./cons. rate	—	Actor’s average execution time
	Level of convergence	4	—
	Level of divergence	4	—
	Average degree of concurrency	—	Token prod./cons. rate
System	Frame per second (fps)	—	—

The important core-level metrics are as follows.

- *Actor’s number of instructions:* a decomposed actor should have a functionality which can be described in 1000 instructions (limited by a single BRAM capacity).
- *Actor’s average execution time:* this is a measure of average time needed to compute output tokens after reading input tokens. The reciprocal of actor execution time is its throughput which is a measure of the actual flow of tokens into a core in terms of bits per second.
- *Core code efficiency:* this is a measure of code efficiency in terms of the ratio of ALU instructions to non-ALU instructions. Non-ALU instructions are mainly token read and write from/to external FIFOs and NOP instructions.
- *Peak register usage:* this is a measurement of the maximum number of registers of local memory used by an actor including input, intermediate and output variables in a single iteration. The current architecture register limit is 32.
- *Core bandwidth:* the theoretical maximum data rate achieved by a core which is directly proportional to the ratio of the number of input tokens required by the core to the number of instructions.

The important network-level metrics are as follows.

- *Core utilisation:* multi-core processor array is made up of 4×*C* interconnected IPPro cores, where *C* is the number of columns. Each column is locally connected to the next in the PL before passing its results back up to the host (ARM) via the AXI bus. Mapping may not utilise all 4 cores in each column of the data-path.
- *Token production/consumption rate:* this factor defines the dynamics of memory requirements on the interconnect and workload division between actors and is dependent on how the high level algorithm has been decomposed. This is the rate at which tokens are produced or consumed by an actor over a period of time.
- *Level of convergence:* this is a measure of the maximum number of cores outputs that are connected to a single consumer input through the interconnect. Considering the current interconnect, the consuming core can only receive data from a maximum of four producing cores.
- *Level of divergence:* this is similar to level of convergence but is a measure of the maximum number of consuming cores that are connected to a single core output through the interconnect.
- *Average degree of concurrency:* this is a measure of average number of actors running ALU operations concurrently. Since the soft-cores run their code sequentially similar to the conventional CPUs, the real performance improvement of this design is the parallel execution of multiple sequential runs.

The important system-level metric is as follows.

- *Frame per second (fps):* a high level analysis will report on this for a particular algorithm and includes estimated delays associated with the controllers and host CPU management software. If a system cannot meet the required fps, it will be deemed as a failure. As discussed earlier, Eq. 1 gives an estimation of its value to be used in partitioning/decomposition processes.

### Compiler Infrastructure

Our developed compiler infrastructure stage of dataflow framework, shown in Fig. 5, is composed of three major steps. The first step investigates the xdf dataflow network file generated in the decomposition/SIMD application stage and assigns the actors to the processors on the network and keeps a record of the settings for each actor to communicate with the other ones to establish the data streams. Also an actor should send the tokens in a predefined order to the target actors. The target actors also expect the tokens in that order. This issue is resolved in this first step of compilation process. The second step of the compilation is the conversion of each actor’s RVC-CAL code to IPPro assembly code. Target specific optimisations are also carried out at this level. For instance, the IPPro is able to process MUL and ADD operations in a single clock cycle. The compiler will replace consecutive MUL and ADD operations with a single MULADD operation.

As will be explained later, a Zynq device has been used as a target in our project. The compiler is responsible to generate the settings for the AXI Lite Registers based on the algorithm, in order to help the controllers distribute the tokens among the cores and gather the produced results. Also some C control functions have been developed which depending on the algorithm, manage the implementation of the design. The parameters required by these functions are also generated by this compiler.

## Case Study: Histogram of Oriented Gradients Algorithm (HOG)

This section presents the implementation of an application use case, namely HOG, in order to evaluate the proposed multi-core IPPro architecture as a programmable acceleration solution. The development board chosen for the HOG implementation is ZedBoard which features a XC7Z020 Zynq device and contains a number of peripheral interfaces. Industry standard AXI interfaces provide high bandwidth, low latency connections between the two parts of the device. The XC7Z020 Zynq is one of the smaller devices in the Zynq-7000 range, and it is based on the Artix-7 logic fabric, with a capacity of 13,300 logic slices, 220 DSP48E1s, and 140 Block RAMs (BRAMS). Additionally, the Zynq device interfaces to a 256Mbit flash memory and 512MB DDR3 memory, both of which are found on the board. There are two oscillator clock sources, one operating at 100MHz, and the other at 33.33MHz.

**Figure 12:**
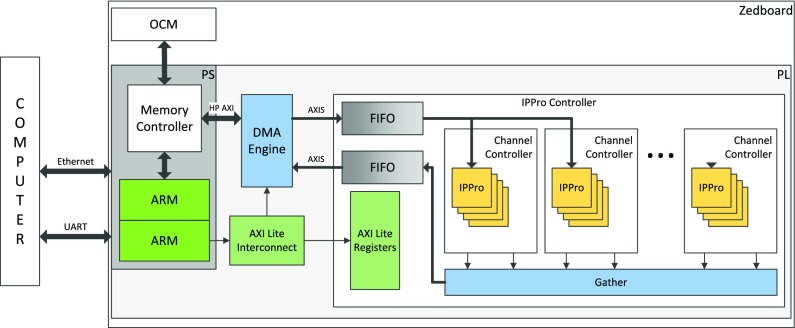
Our high level system architecture.

Our generic high level system architecture for the proposed solution is shown in Fig. 12. A desktop computer is used for testing purposes. This computer can send commands to the PS using the UART connection, which gives console access to the Linux operating system. The Ethernet connection can be used for larger data transfers to/from the Zedboard such as image data.

The data communication between the PS and the PL is provided by the HP ports since this gives a much higher throughput than the GP ports. The GP ports are also used, but only to provide read/write access to the AXI Lite Register space inside the DMA Engine. This register space contains registers which store the address and size of the current data transfer, which allows the ARM processors to start a data transfer between the PL and the Off Chip Memory (OCM) by writing to these registers. The IPPro controller also contains an AXI Lite Register space which allows the ARM processors to control the function performed by the controller among others.

To transfer the image data from the PS to the PL, the OCM is mapped by the ARM processors allowing the image to be copied across. This data can now be accessed by both sections of the Zynq; the PS accesses the RAM through the memory controller, which also gives the DMA Engine access to the RAM through the HP ports. On the PL side of the DMA Engine, the AXI-Streaming (AXIS) interface is used; this is a unidirectional interface standard between two points, so to provide access back to the OCM, a second AXIS port is used. It is necessary to insert FIFO buffers onto the AXIS data path to allow the DMA Engine to operate at a higher throughput. An IPPro controller will receive the data on one of these AXIS ports and output the processed image on the other port. A Linux based operating system is running on the ARM processors.

AXI Lite Registers in the IPPro controller allow the ARM processors to control which operation is performed by the controller. This allows a program running on the ARM processor to read/write to registers inside the controller, which means it can choose which operation to perform by writing a control word into a register, or check the status of the controller by reading from a different address. The AXI Lite Registers currently implemented in the controller are as follows:

- Control: allows the program to set the size of the image and control which operation to be performed; in our current implementation, the controller performs some limited operations such as a 3x3 or 5x5 kernel application on a colour or gray-scale image (Read/Write).
- Status: allows the program to read whether the controller is currently running or there has been an error with the command (Read Only).
- Go: allows the program to start an operation defined by the current value in the control register (Write Only).

**Figure 13:**
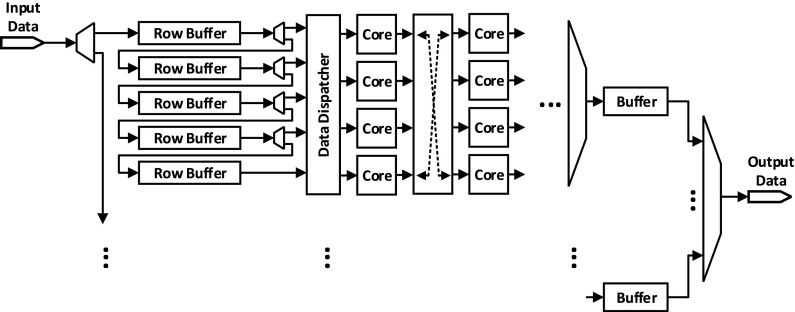
Input/output controllers.

The IPPro controller controls a channel controller and a gather module, each having their own state machine and receiving signals from the top level controller for proper operation. Figure 13 shows a simplified hardware architecture for the input and output controllers.

Channel controller is used to control the operations of a network of 4 × 4 array of cores, where multiple channel controllers are used when implementing SIMD operations. At the top level, the IPPro controller dispatches the input data to one or more channel controllers for SIMD implementation. For each of these channel controllers, the input data could be a 16-bit value, which allows 16-bit grayscale values to be used, or for RGB images, the data will only be 8-bit. In our current implementation, the channel controllers contain five row buffers to store the input data so that the required window can be presented to the correct IPPro; this supports maximum of 5-row windows. Dispatching this input data to the appropriate IPPro is handled by a state machine.

Because the output data is coming from multiple IPPros, a gather module is required to receive data from the processors in turn and send this data to the output port. In order to achieve the required functionality, the gather module uses a state machine to control the operations. It contains two counts to control which channel controller, and which IPPro within the channel controller the data is coming from.

Most of the design units explained in this paper have been fully implemented and the initial version of the others are available. The partitioning/decomposition unit is planned and an early version is currently working. The compiler from RVC-CAL to IPPro assembly has been fully developed and an initial version of the PS/PL implementation also works. A case study was used then to validate the operation and to demonstrate the applicability of our approach.

The case study presented is the HOG algorithm which is a well known algorithm used for human detection by utilising the gradient orientation [12]. Details of the design are given in [18] but this section concentrates on how it is implemented and explored using our design flow. The application of the main steps to HOG are discussed next.

### Partitioning and Decomposition

**Figure 14:**
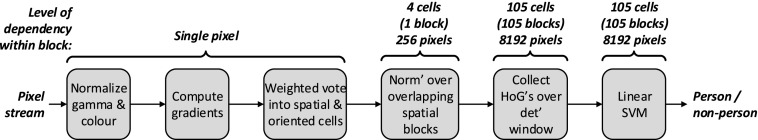
HOG data dependencies [18].

As mentioned in Section 4.1, the behavioural code is partitioned/decomposed into units such that the data dependency between units is kept low and the required performance is met. The common small image processing functions are often the best candidates to be detected as individual actors. The high-level behavioural description of the HOG algorithm includes the six functional units shown in Fig. 14. The decomposition tool detects these explicit functions and breaks the code into these units in order to exploit the low data dependency between them.

The HOG algorithm converts the pixel intensity information to the gradient information, where gradients consist of magnitude and direction as per the first two stages of Fig. 14. Each of the detection windows is divided into cells which are translated into histograms representing the gradients in the cell as per the third stage. The histograms from multiple cells are then normalised with each other to generate a vector as shown in the fourth stage. Collation of the normalised vectors over the detection window in stage five produces the HOG descriptor. In the final stage, a pre-trained off-line Support Vector Machine (SVM) receives the vectors and multiplies with its set weights to achieve the human detection chain.

Three of the six functional blocks, ‘Compute gradients’, ‘Weighted vote into spatial and orientation cells, and Normalise over overlapping spatial blocks’ were targetted to be accelerated using the PL. The software/hardware partitioning tool offloads the non-native IPPro functions to the Zynq ARM cores as they mostly require memory re-organisation. As mentioned earlier, doing this at the host level is the most efficient method as it avoids passing large volumes of data between PL and the PS, thereby avoiding costly transport delays.

**Figure 15:**
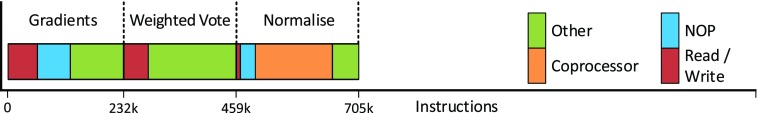
Instruction profile for a single detection window [18].

The instruction profile and cumulative number of instructions to generate the HOG descriptors required for a single detection window is shown in Fig. 15. To generate HOG descriptors for one HD frame at single scaling and no overlapping, 270 detection windows are needed in this implementation. Input data in this instance is 8-bit grey-scale. The number of instructions in this table are the total instructions required to achieve a detection window HOG descriptor for each stage. The total instructions for each function is measured by considering the number of instructions in every actor of that function and the number of actors’ iterations per detection window.

The IPPro architecture used here has a local memory size of 64×16-bit and includes the division instruction in its ISA. We implement the division as a parallel coprocessor in order to provide a speed up while allowing the IPPro core to continue its operation [18].

**Figure 16:**
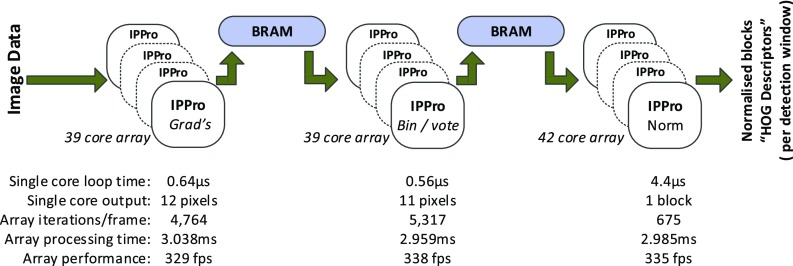
120 IPPro core architecture generating HOG descriptors from a stream of gamma corrected pixels - performance quoted per HD frame generated [18].

With the task-level parallelism of the three functional units, data-level parallelism is also achieved by the decomposition tool by creating core instances with the same instruction code each handling a different window of the frame. With 120 total number of the IPPro cores, organised as illustrated in Fig. 16, the core-level constraints are satisfied. For this specific design, the network-level constraints were not applied as the core-level and system-level measurements were the main focus. These measurements are reported in Table 4. The HOG figures in this table are from the 64×16-bit register file, optimised input window aspect ratio and division coprocessor included. The ‘Normalise’ function is a special case as it violates the constraint of the number of instructions and is handled through manual methods to demonstrate the principles and the concept.

**Table 4:** The metrics used in decomposition phase of HOG algorithm.

Level	Metric	Max	HOG
Core	Actor’s no. of instructions	1000	Grads: 338
			Binning: 295
			Normalise: 2344
	Actor’s average execution time	—	Grads: 64 *μ*s
			Binning: 56 *μ*s
			Normalise: 4.4 *μ*s
	Core code efficiency	<100 %	Grads: 46 %
			Binning: 78 %
			Normalise: 84 %
	Peak register usage	64	Grads: 60
			Binning: 60
			Normalise: 36
	Core bandwidth (Gbs ^−1^)	4.2	Grads: 1.5
			Binning: 1.58
			Normalise: 0.13
Network	Core utilisation	100 %	—
	Token prod./cons. rate	—	—
	Level of convergence	—	—
	Level of divergence	—	—
	Average degree of concurrency	—	—
System	Frame per second (fps)	—	329 (for single scale)

### Compilation from RVC-CAL to IPPro Instructions

The xdf dataflow network file generated in decomposition stage along with each actor’s RVC-CAL code are processed by the compilation tool to generate IPPro instructions. The xdf file processing maps the actors onto cores and allows the actors to configure the network properly. Depending on the algorithm, the compiler also generates the settings for the AXI Lite Registers and the parameters required by C control functions running on ARM.

### Implementation

This design maps data flow control and three functional units onto an ARM core. The other three compute-intensive functional units discussed above are mapped on multi-core processing data path realised on PL. The design implementation approves the system fps metric determined in the decomposition stage.

The resource usage and performance metrics for this design with comparison to other recent implementations are shown in Table 5. A performance of 328 fps can be achieved by this design approach.

**Table 5:** Resources usage of 120 core IPPro design and recent FPGA implementations [18].

Ref	Device	Clock	LUTs	DSPs	BRAMs	Resolution	fps
Our	XC7Z020	530 MHz	47,720	120	120	1920 × 1080	328
[15]	XC5VFX200T	270 MHz	3,924	12	26	1920 × 1080	64
[20]	XC6VLX760	150 MHz	92,477	191	95	640 × 480	68
[21]	XC5VLX50	44.85 MHz	17,383	no data	36	640 × 480	112

Two versions of the functional blocks were explored, a hand-coded VHDL description which took 40 days to code and which was validated using VHDL-based tools and the other, an IPPro implementation. The IPPro design was implemented before the compiler was implemented and it took 10 days to generate the code and test. With the compiler, it was implemented in less than a day and iterated in a matter of minutes. The design time savings are a result of the deterministic coding and behaviour of the IPPro which allow the user to compile the design quickly and accurately calculate the functionality on a cycle by cycle basis. Whilst this is not scientific, it gives some indication of design time saving.

## Conclusion

This paper presents a high level dataflow framework for soft-core processors on FPGA for image processing applications. We have demonstrated the potential of replacing the conventional hardware design route for FPGAs with the use of custom designed soft-core processors and programming these processors with a dataflow-based design approach. The idea of decomposing and translating dataflow programs written in RVC-CAL to IPPro assembly is presented through a case study, the HOG algorithm and it is shown how the design approach reduces design time and effort.

The overall design framework with limited optimisations and limited memory access is currently operating and many target based optimisations and profiling are done to improve the mapping of future designs and thus improve efficiency, allowing us to get near the same performance of any hand-crafted design. Another important aspect of the design of the processors and the framework is the programmable interconnect which will increase the flexibility and also the capability to map more complex algorithm onto the platform. However more flexible hardware will introduce new challenges to the framework to reflect and optimise the use of resources.
